# Case Report: Microfragmented Adipose Tissue Drug Delivery in Canine Mesothelioma: A Case Report on Safety, Feasibility, and Clinical Findings

**DOI:** 10.3389/fvets.2020.585427

**Published:** 2021-01-14

**Authors:** Offer Zeira, Erica Ghezzi, Letizia Pettinari, Valentina Re, Davide M. Lupi, Silvia L. Benali, Simone Borgonovo, Giulio Alessandri, Francesco Petrella, Rita Paroni, Michele Dei Cas, Carlo Tremolada, Valentina Coccè, Augusto Pessina

**Affiliations:** ^1^Department of Stem Cells and Regenerative Medicine, San Michele Veterinary Hospital, Tavazzano con Villavesco, Italy; ^2^Laboratorio La Vallonea, Milan, Italy; ^3^Clinica Veterinaria Crema, Crema, Italy; ^4^Department of Cerebrovascular Diseases, Istituto di Ricovero e Cura a Carattere Scientifico, Besta Neurological Institute, Milan, Italy; ^5^Centro di Ricerca Coordinato StaMeTec, Department of Biomedical, Surgical and Dental Sciences, University of Milan, Milan, Italy; ^6^Department of Stem Cells and Regenerative Medicine, Istituto di Ricovero e Cura a Carattere Scientifico, European Institute of Oncology, Milan, Italy; ^7^Centro di Ricerca Coordinato StaMeTec, Department of Oncology and Emato-Oncology, University of Milan, Milan, Italy; ^8^Department of Health Sciences, Università Degli Studi di Milano, Milan, Italy; ^9^Department of Stem Cells and Regenerative Medicine, Istituto Image, Milan, Italy

**Keywords:** mesothelioma, paclitaxel, adipose tissue, dog, drug delivery

## Abstract

Mesothelioma is a rare lethal tumor of dogs and humans involving cavities of the body. Dogs are considered a model for new drugs and therapeutic methods since they present spontaneous diseases similar to humans. Microfragmented adipose tissue (MFAT) uploaded by paclitaxel (PTX) is a drug delivery medium providing slow release of chemotherapic drugs. A dog affected by pleural, pericardial, and peritoneal mesothelioma was treated by 17 intracavitary ultrasound-guided injections of MFAT-PTX over 22 months. A long-lasting improvement of general conditions was observed, treatment was well-tolerated, and no toxicity or hypersensitivity was reported. Pharmacokinetic (PK) data indicated low drug localization in the circulatory system and a tendency to enter or remain in the extravascular compartments of the body. Indeed, low levels of free-circulating drugs for a short time produced low toxicity, whereas, a higher intracavitary PTX concentration can have major pharmacological efficacy. To our knowledge, this is the first time that mesothelioma has been treated using such a procedure, and this should be considered as a novel therapeutic approach. The low systemic absorption suggests the possible role of MFAT-PTX for loco-regional/intratumoral therapy also useful in other types of tumors, and further investigation is warranted.

## Background

Mesothelioma is a rare neoplasm of dogs and humans affecting the lining epithelial cells of the coelomic cavities of the body. It is a chemoresistant tumor and actually has no effective therapeutic strategies. In human medicine, around 80% of this tumor occurs in men, median survival is 8–18 months, and the survival rate is < 12% in the 5 years after diagnosis. In veterinary medicine, no sex predilection is reported. Median survival for untreated dogs with mesothelioma is difficult to assess since they are often euthanatized following diagnosis. Various studies of treated dogs (surgery, intracavitary, and intravenous chemotherapy) report survival time from 2 to 13 months (1). In both humans and dogs, the tumor may involve cavities such as thorax, abdomen, pericardial sac, and the vaginal tunics of the scrotum.

The domestic dog is considered to be an important animal model for the evaluation of new drugs and therapeutic methods since it presents spontaneous diseases very much similar to human oncology (2, 3), including mesothelioma. Microfragmented adipose tissue (MFAT) is a potential drug delivery medium that may provide a slow release of chemotherapic drugs contiguous to the tumor (4). MFAT consists of stromal vascular fraction, pericytes, and adipose stromal stem cells, and it is known to have trophic, mitogenic, anti-scarring, anti-apoptotic, immunomodulatory (5), and antimicrobial actions produced by a large number of bioactive elements, growth factors, and cytokines (5–7).

In order to obtain MFAT, we employed a commercially available, enzyme-free technology (Lipogems) able to harvest micro-fragmented fat preparation. This technology reduces the size of the adipose tissue clusters by means of mild mechanical forces while eliminating pro-inflammatory oil and blood residue. The technique is gentle and provides micro-fragmented fat in a short time (15–20′) without expansion and/or enzymatic treatment (8, 9). Paclitaxel (PTX, Taxol®) is an anticancer chemotherapy drug that isclassified as a “plant alkaloid” and is used in various types of solid tumors in human medicine. Although the hypersensitivity and side effect related to the toxicity of paclitaxel and cosolvents are well-known (10), numerous studies have suggested the efficacy of paclitaxel in different types of tumors using both systemic and subcutaneous injection (11–14). Whereas, intracavitary chemotherapy in veterinary mesothelioma was described using platinum-based drugs (15), paclitaxel is not considered the therapy of choice for mesothelioma. In agreement with new approaches oriented to verify and validate new paclitaxel formulations, the aim of our study is to verify the safety, feasibility, and efficacy of intracavitary administration of paclitaxel-loaded microfragmented adipose tissue in mesothelioma in dogs.

## Case Presentation

### Patient

A 6-year-old, mixed-breed, 24-kg, neutered dog, with a 2-month history of a progressive weakness, loss of appetite, productive cough, abdominal distension, and difficulty in breathing, was selected for the treatment. Complete blood count (CBC) and biochemical tests were assessed. Thoracic radiographs detected severe pleural effusion and bilateral middle and caudal lung lobe collapse. Abdominal radiographs and ultrasound also evidenced severe effusion. Serosanguinous fluid was removed from the pleural and abdominal spaces, underwent cytological examination, and showed high malignant characters (anisocytosis and anisokaryosis). Ultrasound-guided biopsies were taken from the pleura, pericardium, and peritoneum, and histological and immunohistochemical diagnoses were performed; the final diagnosis was of mesothelioma.

### Paclitaxel-Loaded Adipose Tissue (MFAT-PTX) Preparation

Autologous adipose tissue, obtained by lipoaspirate from the dog's lumbar flanks, was microfragmented by using a microfragmentation device (Lipogems, Italy) as previously described (16). This procedure allows minimal manipulation without the use of enzymatic procedures, and microfragmented samples can be also cryopreserved or used as a scaffold for paclitaxel (MFAT-PTX). For our study, fresh aliquots of microfragmented adipose tissue were loaded with PTX by adding the drug at a concentration of 1 mg/ml and stirring the mixture for 30 min before use as previously reported (4).

### Chemotherapeutic Protocol

Treatment protocol consisted of ultrasound-guided administration into the abdominal and thoracic cavity of 7 ml of MFAT-PTX (1 mg/ml) corresponding to 0.29 mg/kg (0.35 mg/m^2^) of free drug. First administration (T0) was done 15 days after presentation. Over the course of 22 months, the dog underwent 17 treatments (both intrathoracic and intra-abdominal), with an average of a treatment every 38 days (shortest interval 14 days, longest 70 days).

**Figure 1:**
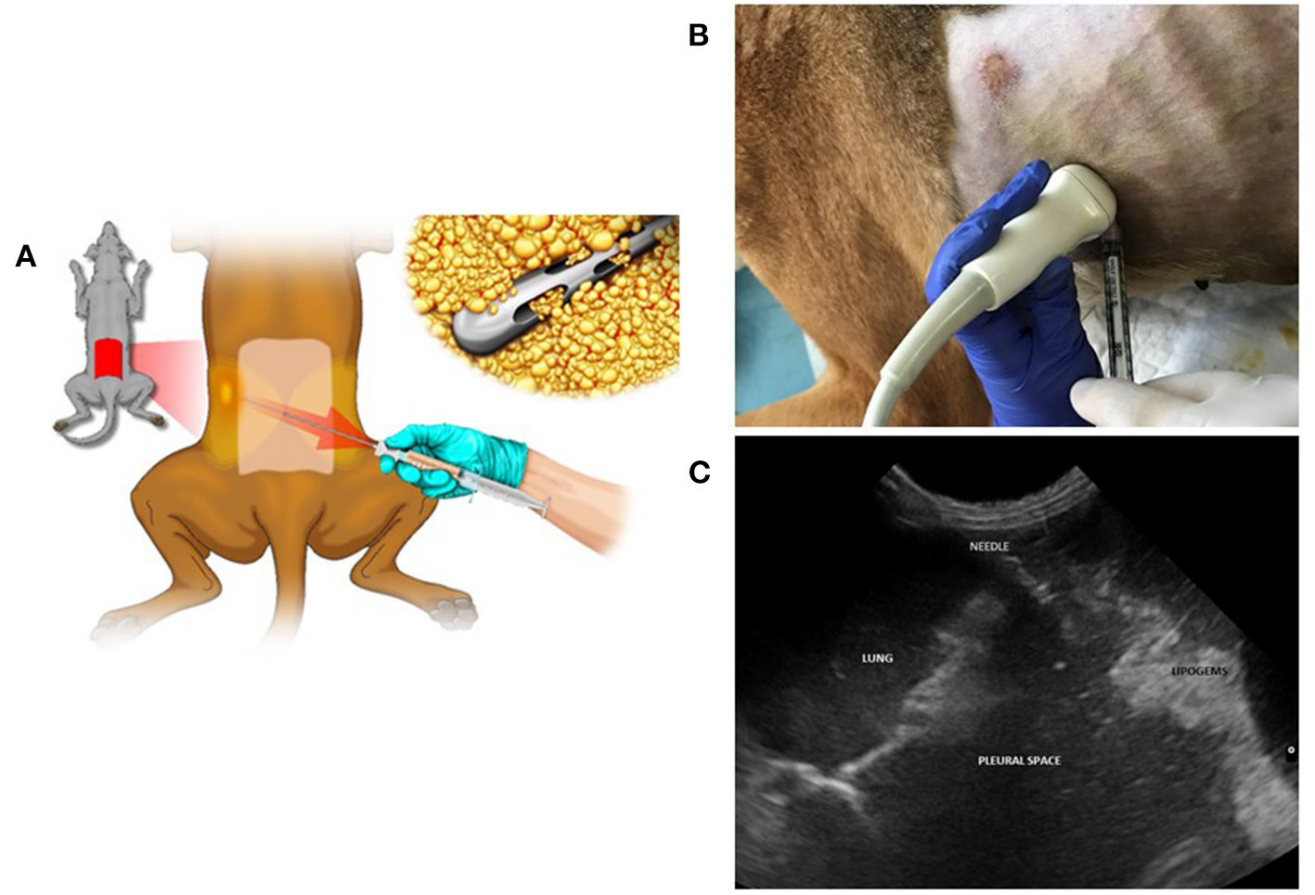
Lipoaspirate collection and ultrasound-guided intrathoracic treatment with MFATPTX. **(A)** Lipoaspirate collection from the dog's lumbar flanks. **(B)** Procedure of treatment that was initiated by drainage of abdomen and chest from the exudate fluid followed by the injection of 3 ml of MFAT-PTX intraperitoneally + 2 ml, respectively, into the right and the left pleural spaces. **(C)** Ultra sound view.

The procedure is initiated by drainage of the abdomen and chest from the exudate fluid followed by injection of 3 ml of MFAT-PTX intraperitoneally + 2 ml, respectively, into the right and left pleural spaces (Figure 1). Clinical outcomes were monitored at the time of each treatment and documented by thorax radiographs, abdominal ultrasound, clinical examination, and blood counts. Special attention was given to eventual adverse effects.

### Pharmacokinetics (PK) Study

The minimal parameters of PK [C_max_, T_max_, T_1/2_, volume of distribution (Vd), and area under curve (AUC)] were evaluated by checking the amount of drug in the blood 30 min and 2, 4, and 8 h after the first treatment of the MFAT-PTX. At the 10th treatment (T10), the residual amount of the drug was also measured in the pleura and pericardium biopsies. The amount of PTX was evaluated by liquid chromatography-coupled mass spectrometry (LC-MS/MS) analysis as already reported (4). Briefly, blood (100 μL) and tissues (about 50 mg) were added in internal standard (25 μl of paclitaxel D5 10 μg/ml) and PTX was extracted by 1 mL of a mixture methanol/isopropanol (6:4, v:v). Dry extracts were redissolved with 200 μl of acetonitrile and 10 μl injected in LC-MS/MS. The details for instrumental conditions were already described elsewhere and kept essentially unaltered (4).

### Clinical Assessment

No major short- or long-term adverse effects were registered. Complete blood counts and biochemistry performed each month did not show abnormalities. In particular, during the whole treatment leukocytes and platelet counts in the peripheral blood indicated constantly normal values, suggesting an absence of systemic myelotoxicity. All phases of the treatments were feasible. In order to avoid iatrogenic damage to the internal organs, major attention was given to the intracavity administration when a low amount of effusion was present.

The dog presented rapid improvement in its general conditions after each exudate fluid drainage, followed by the intracavitary administration. This effect lasts for an average period of 30–40 days.

Notably, in the first 15 months of treatment the intervals between treatments were longer (average 52 days) while in the last 7 months the intervals between treatments were shorter (average 24 days).

The patient presented good clinical conditions during most of this period. During the final few days before each treatment, exudate formation became visible, and the clinical conditions worsened.

Evaluation of the clinical status was based on the periodical veterinarian controls in which exudate fluid formation was assessed by ultrasound, radiographs, and computed tomography scan (CT) together with breathing quality. Both parameters presented clear amelioration. Notably, the abdominal exudate production completely ceased after the fourth treatment. In addition, the owner's evaluation was taken into consideration, as they had knowledge of the dog's “normal” vs. “abnormal” behavior. The owner reported a good quality of life. The dog was able to play with other dogs, go up the stairs, and coughed rarely, breathed normally most of the time, and had a good appetite.

### Imaging Diagnostic Assessment

**Figure 2:**
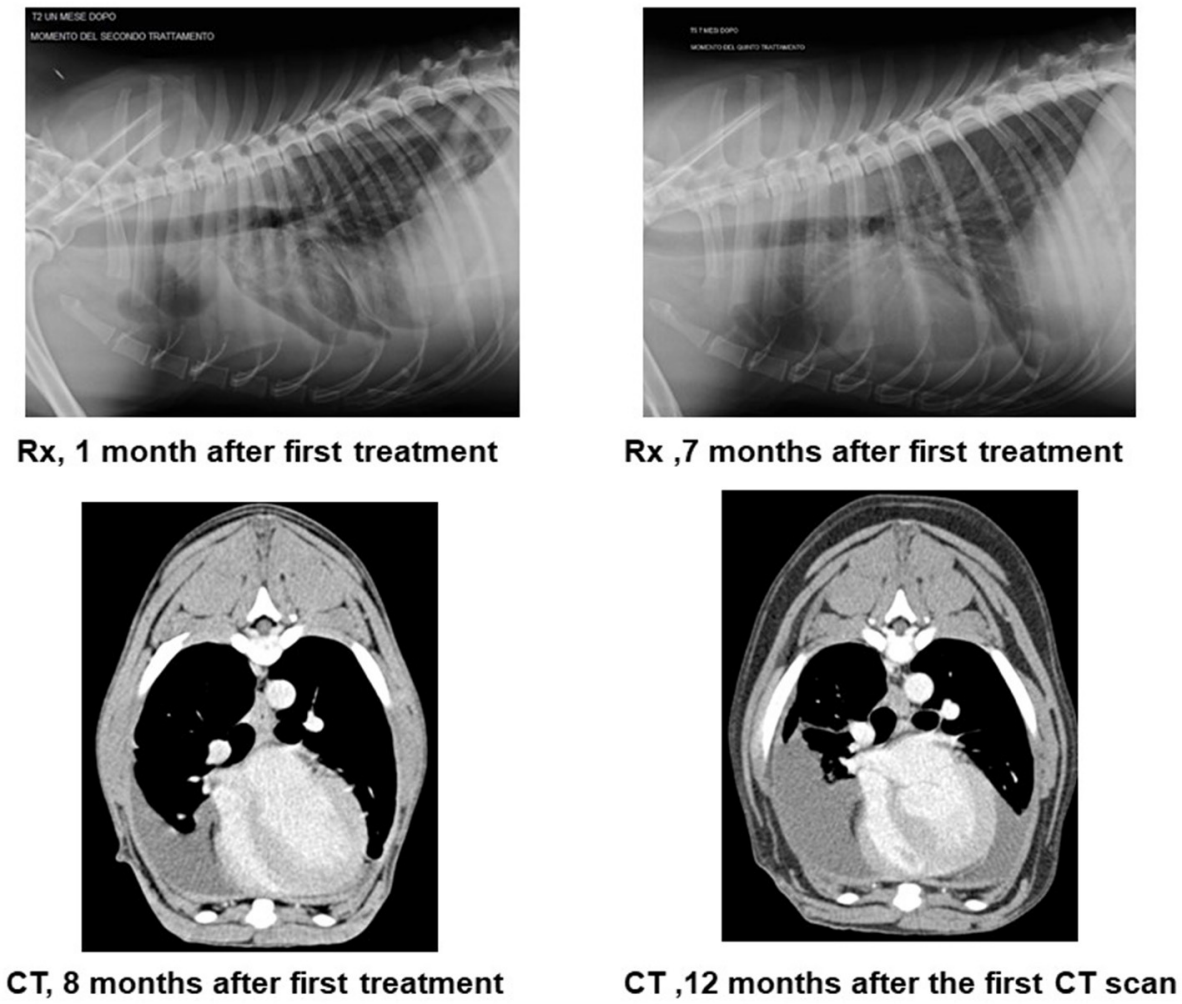
Radiography and computed tomography scan (CT) along the treatment. The figure reports the post-treatment thoracic radiographs (Rx) performed once a month in the first 6 months. They showed progressive reduction of the severe pleural effusion. The computed tomography scan (CT) shows the first CT scan 8 months after first treatment. Transversal view of the soft tissue window, and post-contrast right side effusion. The control 12 months after the first CT scan shows a bilateral pleural effusion mildly increased.

Post-treatment thoracic radiographs, performed once a month in the first 6 months, showed progressive reduction of the severe pleural effusion (Figure 2). The following radiographs, every 3 months, evidenced only mild differences in the thoracic effusion quantity while in the abdominal cavity no liquid could be detected. The first thorax and abdominal CT scan, pre- and post-contrast medium, performed 8 months after the initial treatment evidenced mild to moderate pleural effusion, rare abdominal effusion, pleura and peritoneum reactivity, and loco-regional lymphadenopathy. A control CT after 12 months showed only mild difference (Figure 2).

### Pathological Assessment

**Figure 3:**
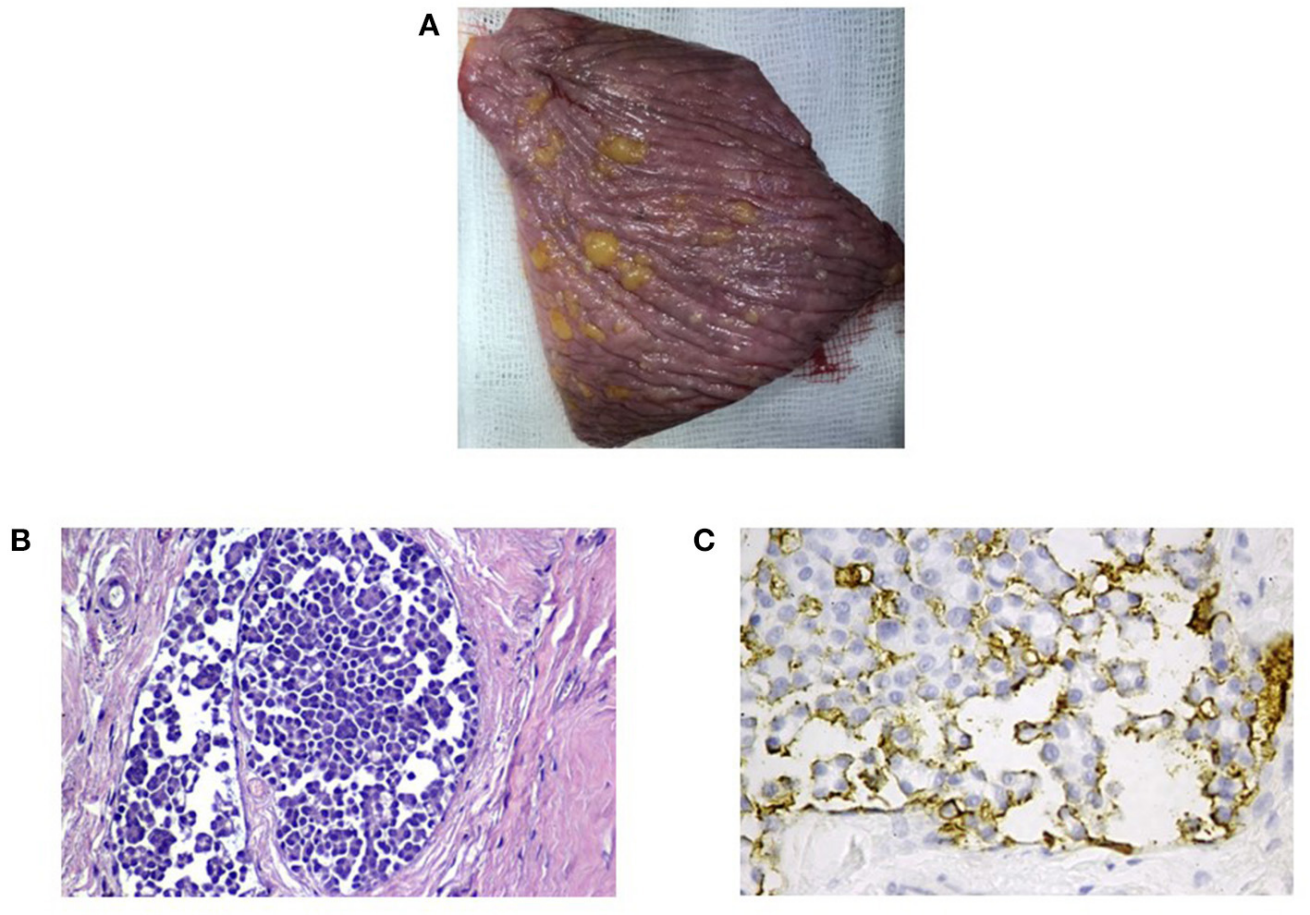
Macroscopic and histological analysis of pleura. **(A)** The macroscopic analysis shows the presence on the pleural surfaces isles of yellowish fatty material, identified as MFAT-PTX (last administration 2 weeks previously). **(B,C)** Histological section of pleura (hematoxylin and eosin stain) shows neoplastic cells within the lumen of dilated vessel. Immunohistochemistry for the HBME–mesothelioma marker shows moderate to intense membranous immunolabeling of the tumor cells with apical membrane accentuation.

The patient was euthanized after 22 months due to worsening of his clinical conditions and underwent a complete post-mortem exam. Macroscopically all the pleural surfaces presented isles of yellowish fatty material, identified as MFAT-PTX (last administration 2 weeks previously) (Figure 3). Cytological smears of pleural and peritoneal effusion were evaluated, presenting overlapping findings: hematic background with scattered eosinophilic material, good cellularity, and mixed cellular population; and presence of numerous cells in clusters of small/medium size, having variable cytoplasm nucleus/cytoplasm ratios, slightly basophilic cytoplasm with occasional vacuolization, round/oval central or paracentral nucleus with coarse chromatin, and prominent central nucleolus. The cells present moderate to severe malignancy characters, including anisocytosis and anisokaryosis and presence of occasional leukocytes.

A biopsy of pericardium was fixed in 10% buffered formalin, embedded in paraffin, and stained routinely with hematoxylin and eosin. Immunohistochemistry was performed on paraffin sections (30 μm) placed on Superfrost Plus slides (Superfrost® Plus) with an automated immunostainer (Discovery Ultra-Roche). The primary antibody was a monoclonal anti-mouse mesothelial cell clone HBME-1 (Dako cod M3505) diluted 1:100 and incubated at room temperature for 20 min.

Histopathological sections of pericardium and pleura were examined with focal evidence of a neoplastic cellular component within the pleura engorging dilated vessels. Cells were round to polyhedral and arranged in small groups or micropapillae. The nucleus had a cytoplasmic ratio that was intermediate. The cytoplasm moderate and eosinophilic, and the nucleus was round to oval with 1–2 nucleoli. Anisokaryosis was marked and anisocytosis moderate. Mitosis were 0–2 in 10 high-power fields (400 ×). Immunohistochemistry for the HBME–mesothelioma marker confirmed the mesothelial origin of the cells (Figure 3).

### Pharmacokinetics (PK) Assessment

**Figure 4:**
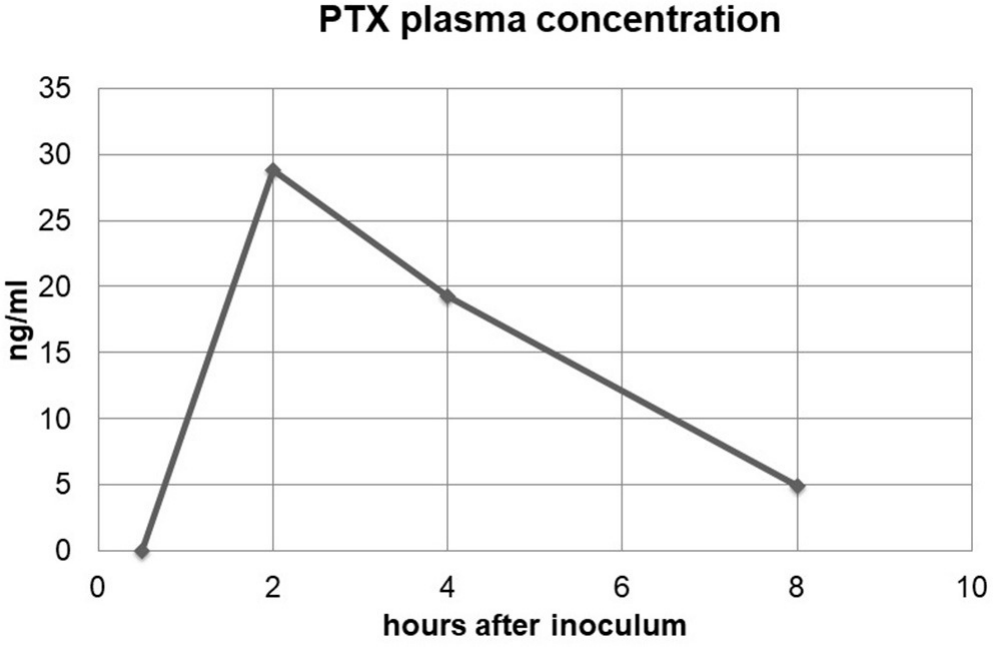
Plasmatic concentration of paclitaxel (PTX). The figure shows the plasmatic concentration of PTX after the first treatment with MFAT-PTX as reported in the chemotherapeutic protocol. The amount of PTX was checked in the blood after 30 min and 2, 4, and 8 h.

After treatment with MFAT-PTX, no drug was detected in the blood at 30 min. At 2, 4, and 8 h, the amounts detected in plasma were 28.8, 19.25, and 4.89 ng/ml, respectively (Figure 4). C_max_ was 28.8 ng/ml, T_max_ = 2 h, and the half-time (T_1/2_) = 5 h. Based on the dose of PTX injected and the plasmatic C_max_, we estimate an apparent volume of distribution at 2 h (Vd_2h_) of 243 liters. The calculation of plasmatic AUC 1–8 h demonstrated that MFAT-PTX treatment makes available the drug in plasma with a value of 117 ng^*^h/ml. The residual amount in pleura and pericardium measured at 30 days after the 10° treatment by HPLC suggested the presence of 3.6 and 13.3 ng/g, respectively, which, by considering the weight of these tissues, can be evaluated as a residual drug of about 2.16 μg in the pleura and 3.99 μg in the pericardium.

## Discussion

Mesothelioma is a fatal disease in both dogs and humans, and new effective therapeutic strategies are needed (17). Our data suggest that localized delivery of microfragmented adipose tissue (MFAT) uploaded by paclitaxel (MFAT-PTX) directly into the peritoneal and thoracic cavity is feasible. Administration in average intervals of 38 days was well-tolerated in the dog, and no unique toxicity or hypersensitivity was noted. This result is of particular interest. Due to the low aqueous solubility of paclitaxel, Taxol® formulations include Cremophor® (polyoxyethylated castor oil) and ethanol as an excipient. Such formulations overcome poor solubilization of paclitaxel for parenteral use. However, Cremophor®-induced complement activation is believed to be the cause of common hypersensitivity reactions related to Taxol® use in humans and other species (10, 18, 19). The absence of hypersensitivity in our case may be explained by various ways. MFAT is known to have strong immunomodulation activity (9). Another possible explanation is the long intervals between treatments together with the slow release of low doses.

The MFAT-PTX scheme of treatment seems to be able to produce a local, rather long-term, antineoplastic effect without any systemic myelotoxicity. A CT control scan after 12 months showed no major difference in the effusion quantity, pleural and peritoneal reactivity, and lymphadenopathy, which indicates a reduction in tumor progression. The long-lasting improvement in the dog clinical conditions was regularly reported from both veterinarian and owner and is probably due to the slow release of paclitaxel from the microfragmented adipose tissue in the thorax and abdomen, as already reported (4). As far as the thoracic cavity is concerned, from our data in the first 15 months of treatment, the intervals between local administrations were longer and became progressively shorter in the following months (from average of 52–24 days). The need to shorten the intervals in the last months of treatment was due to a slow and progressive increase in the thoracic effusion. On the other hand, in the peritoneal space, effusion quantity was very much reduced within 6 months after the initial treatment and remained such until the last treatment. This observation is important since the dose of paclitaxel and MFAT volume remained the same in all treatments. It may find an explanation by assuming that the tumor developed some grade of resistance, but it does not explain why the peritoneal compartment behaved in a different way, maintaining a very low level of effusion during the whole period. In order to answer this query, a much larger number of patients are warranted.

The PK data showed a very high value of Vd indicating that, after treatment, the drug localization in the circulatory system is low and that the drug has a propensity to enter or remain in the extravascular compartments of the body. This is compatible with the chemical structure of PTX, which is a lipophilic molecule. It is also a consequence of the loco-regional treatment with MFAT-PTX resulting in a more significant distribution of PTX into the areas with higher lipid density.

As known, this parameter together with the above reported gives an index of the systemic drug exposure. The low value is predictive of low systemic toxicity due to the loco-regional treatment producing low levels of free-circulating drug for a short time, whereas, locally, PTX concentration can have pharmacological efficacy. This is also confirmed by the low modulation of blood cell counts during the treatment and by the presence of significant residual amounts of PTX found in both pleura and pericardium 1 month after treatment.

Even though our experience with this therapeutic procedure includes more than one patient, we have decided to limit our report to this single case due to its very intense follow-up and detailed investigation from diagnosis to post-mortem assessment.

To our knowledge, this is the first time that mesothelioma is treated using such a procedure in a dog and should be considered as a novel therapeutic approach for mesothelioma treatment. Furthermore, the lack of systemic absorption after intra-abdominal and intrathoracic administration suggests a possible role of MFAT-PTX for intratumoral therapy. Such a procedure may also be useful in other types of tumors, and further investigation is warranted.

We have used this new procedure in 2 other cases of mesothelioma. Unfortunately, both cases were presented to our hospital in a rather advanced clinical status and died within 1 month for cardiopulmonary failure. However, the same procedure was applied to other 2 patients with tumors such as spleen hemangiosarcoma and ovarian carcinoma with diffuse abdominal involvement. At the present time, results are similar to our mesothelioma case by means of no major short- or long-term adverse effects and overall survival time.

The lack of complications in the dog should be taken into account when considering this treatment in other species, including man. The study of spontaneous, naturally occurring tumors in dogs is a model that provides a valuable role in developing potentially successful, innovative treatment regimens for translational medicine, facilitating the transfer of knowledge from the “bench” to the “bedside.”

## Data Availability Statement

The raw data supporting the conclusions of this article will be made available by the authors, without undue reservation.

## Ethics Statement

The procedures involving MFAT-PTX were performed in accordance with the guidelines defined by the Italian Presidency of the Council of Ministers and following the guidelines published by the General Directory of Animal Health and Veterinary drugs of the Italian Ministry of Health. The owners considered euthanasia for their dog but accepted our proposed treatment as a last possibility. They were thoroughly informed about the entire procedure and signed a formal agreement with the San Michele Veterinary Hospital in acceptance of both anesthesia and therapy. They also accepted that their dog would undergo post-mortem examination. The veterinary hospital followed guidelines established for Good Clinical Practice.

## Author Contributions

OZ, LP, EG, VR, DL, SLB, and SB: medical diagnosis, management of case, and collection of data. RP, MD, and CT: laboratory analysis. AP, EG, VC, GA, and FP: collection of data, writing, and editing of manuscript. AP, VC, and FP: review of final submission. All authors contributed to the article and approved the submitted version.

## Conflict of Interest

CT is the scientific director of Lipogems International. The remaining authors declare that the research was conducted in the absence of any commercial or financial relationships that could be construed as a potential conflict of interest.
